# Annotations

**Published:** 1891-02-28

**Authors:** 


### Annotations.

**Inoculation for Crime.**

The subject of inoculation is persistently dragged forward with reference to physical ills, but has it not also its moral application? In a future age, when there will be no more contagious diseases, because we have spent our childhood in a cheerful series of visits to Koch, Pasteur, and Jenner Institutes—in the age when the poor human will languish, but not die, we foretell that there will be a Crime Inoculation Institute. At present there are some of us who would rather have one disease *in toto* and die thereof, than have all diseases *in petto* and live. But preventive medicine will march ruthlessly over our feelings; and after all, if socialism has by then reduced everything to a dead level, and taken the salt from life, time may as well be spent in institutes as in the common lodging-houses of the State. The copy-books always said it was better to be good than to be happy; so when the tedious age of safety and equality comes, let us be good, let us turn the criminal to account, and be inoculated for crime as well as for disease. First, there might be the Murder Institute; here would be kept the last murderer in strict confinement, nd every day some of his diluted sanguineous fluid would be withdrawn and injected into certain youths. Then, while the virus worked, these youths should be encouraged with blunt knives to attack dummies, till, once safely through the fever, they could be sent forth into the community with the knowledge that never again would they feel disposed to attack their fellow-beings. Then there might be the Gambling Institute, and here small boys should fight and cheat, and play for false coins, till the passion having spent itself the counters were put aside for the next batch of patients, and the cured were sent forth warranted never to gamble again. In the same way a mild form of theft, blasphemy, intemperance, etc., might be given to each child, and so prevent these sins from having any hold in after life. Think of the just punishment to the criminal to be thus ever supplying an antidote to his crime; and think of the benefit to the human race. All the schemes of the Socialists, the New Fellowship, and General Booth are not a patch on this one for the utter extermination of vice and the positive triumph of virtue. It sounds excessively dreary, we admit. There would be no baccarat scandals, no Whitechapel murders, no Egyptian campaigns. There would be no more of the old Viking passion in us; we should no longer enjoy the novels of Scott and Kingsley and Rider Haggard. Pitifully tame and colourless will be the age of safety and equality; but we have no more faith in the dreams of others than in our own, and when our Crime Inoculation Institute is started, then shall we expect the fulfilment of "Looking Backward" and the reign of uniformity—and not till then.

**The Seamen's Hospital.**

The seventieth annual Court of the Seamen's Hospital Society (late "Dreadnought") will be memorable as marking a new departure in the history of that society. The meeting was held in the Board-room of the P. and O. Company (122, Leadenhall Street, E.C.), Sir Thomas Sutherland, the chairman of the company, presiding. If there is one class more than another who ought to support the old "Dreadnought" it is undoubtedly the shipowners. For some reason which is not readily understood, the support extended to this charity by shipowners has not been as great as it ought to have been. Mr. Michelli, the present able secretary of the society, has been much concerned at this circumstance, and the meeting at the P. and O. offices is a striking proof of his ability and success. There is no institution in the whole country which appeals more widely to the sympathy of the whole population than the "Dreadnought," because all benefit by the devotion of seamen, and this hospital is free to all sailors, regardless of race, creed, or colour. We congratulate Sir Thomas Sutherland and his company on their support and patronage of this great national charity. Shipowners owe a debt of gratitude to its managers in the past, and we are glad to hear that there is now sound reason to hope that the funds of the institution will greatly benefit.

**Woman's Greatest Right.**

A "Hampshire Governess," who says she is neither old nor even middle-aged, and who, moreover, admits that she can make a comfortable living by her profession, and seems to be pleased with it, contributes a very interesting letter to the *Daily Graphic* on the controversy now being carried on in that paper on "Women and Politics." This Hampshire governess may be young in years, but she is certainly extremely old-fashioned in her ideas and tastes. She actually has the temerity to assert, partly on her own judgment and partly on that of Lady Middleton, that "woman's greatest right is to be a helpmeet to the man she loves." The picture which presents itself to the male mind as it repeats a story of this kind, is that of a large crowd of Roman-nosed and spectacled spinsters, with lank cheeks, glaring eyes, and caustic tongues, all rising up simultaneously in angular indignation, and tearing the governess to pieces. "She wants a husband herself," those virtuous and charitable judges will declare; and being too poor to pay the fees of a matrimonial agency, she secures a cheap advertisement by writing a letter to the *Daily Graphic*. That is surely as sweet and wholesome a picture as Hogarth or Cruickshank ever evolved from the depths of their fertile imaginations! The love of women for women strikes the male creature as all but miraculous: miraculous, that is, in the scientific sense of the word: it is a *semeion* or sign. What will the advanced woman think when she reads that there is at least one man who believes that women ought to have the fullest freedom to be doctors, lawyers, Lord Chancellors or Lady Chancellors, Archbishops of Canterbury or Archbishopesses, and even masters of foxhounds, and who yet in his very inmost heart sympathises with the Hampshire governess, and believes that she is morally, intellectually, philosophically, socially, politically, and practically right; and that her cause is destined to win? Women ought to have, and they will certainly gain, all the freedom men have, but they ought not to unwomanise themselves; it is both ridiculous and unwholesome of them to wish to do so, and they can never succeed. A few of them may make themselves hard, and sour, and hateful. It is a comfort to think that they will make themselves miserable at the same time. Women were made to be as free as men; but they were never made to be in opposition to men. In an ideal world every woman would have one husband, and every man one wife. It is the ideal which should always be aimed at. The standard must be always held aloft, and it must stand for the perfectly true, the perfectly reasonable, and the perfectly right. Whoever wilfully lowers the standard, and consciously aims at less than the best, is, in the court of philosophy, an unscientific person and a fool. There is no truth which has deeper roots in physiology and philosophy than this: That nature made the woman for the man, and the man for the woman. Circumstances may, and do compel many a woman to a solitary life, as they compel many a man; and it is quite possible, and, indeed, an everyday occurrence for such women to be both useful and happy. But when we propound general principles, and when we set out on the path of legislation, we must not think of the few, but of the many; not of the individual, but of the class. It is an absolutely unassailable generalisation that the woman was made for the man, and the man for the woman. It is only as social philosophers and legislators keep this natural and universal fact in view, that their philosophy and their legislation can have any approximation to reason.

